# Severe toxicity following supratherapeutic co‑ingestion of duloxetine and dexzopiclone in a young patient with major depressive disorder: a case report

**DOI:** 10.1186/s13256-026-06736-y

**Published:** 2026-11-06

**Authors:** Wang Jinmin, Lin Jushan, Lin Min, Liao Yunhai, Zheng Chongwen

**Affiliations:** 1 Department of Neurology, The Second People’s Hospital Affiliated to Fujian University of Traditional Chinese Medicine, No.282 Wusi Road, Gulou District, Fuzhou, 350003 People’s Republic of China; 2 Department of Emergency, The Second People’s Hospital Affiliated to Fujian University of Traditional Chinese Medicine, Fuzhou, 350003 People’s Republic of China

**Keywords:** Major depressive disorder, Duloxetine, Dexzopiclone, Drug co-ingestion toxicity, Case report

## Abstract

A 23-year-old female with major depressive disorder (MDD), non-suicidal self-injury and generalized somatic pain was treated with duloxetine hydrochloride enteric-coated capsules, quetiapine fumarate tablets, and aripiprazole orally disintegrating tablets, with significant pain relief. Dexzopiclone tablets were added for insomnia, and all medications were kept by her mother. Despite compliance with medication, the patient had persistent suicidal ideation and secretly ingested 4500 mg duloxetine and 63 mg dexzopiclone on February 18, 2021. She was rushed to hospital 3.5 h later and received emergency treatment including oxygen inhalation, gastric lavage and high-dose vitamin B6/C infusion. Combined with psychological suggestion and cognitive therapy, she recovered and was discharged 12 days later. At the 4.5-year follow-up (September 2025), the patient had discontinued lurasidone and quetiapine, both of which were started during index hospitalization; she demonstrated normal daily functioning with no recurrent suicidal ideation or self-injury. To our knowledge, severe toxicity from co-ingestion of supratherapeutic doses of duloxetine and dexzopiclone has been rarely documented; this report details the clinical management and differential diagnosis strategies for this rare drug-combination toxicity.

**Supplementary Information:**

The online version contains supplementary material available at https://doi.org/10.1186/s13256-026-06736-y.

## Introduction

Major depressive disorder (MDD) is commonly accompanied by somatic pain.A large-scale UK Biobank cross-sectional cohort reported chronic multisite pain occurred in 19.8% of patients with major depression [1], such as back, abdominal and musculoskeletal pain, in addition to core symptoms of depressed mood and anhedonia. Duloxetine, a serotonin-norepinephrine reuptake inhibitor (SNRI) with weak dopamine reuptake inhibitory effects, exerts dual antidepressant and analgesic actions, making it a preferred agent for MDD patients with somatic pain. However, its package insert [2] notes an increased risk of suicidal thinking and behavior (suicidality) in patients aged ≤24 years compared with placebo. It should be noted that the US FDA requires a uniform black-box warning of increased suicidal thoughts and behaviors for all approved medications for major depressive disorder and bipolar depression, not limited to duloxetine as an SNRI agent. Z-drugs, such as dexzopiclone, are third-generation hypnotics for insomnia, with a distinct chemical structure from traditional benzodiazepines. As the mono-enantiomer of zopiclone, it has a faster peak concentration, shorter half-life (5–6 h), and fewer residual effects at conventional therapeutic doses (2–3 mg) [3]. The maximum recommended daily therapeutic dose of duloxetine is 120 mg, with no additional clinical benefit above 60 mg/day [2]. For dexzopiclone, the maximum nightly dose for adults is 3 mg, and is restricted to 2 mg in elderly or severely hepatic-impaired patients [4]. Previous reports have shown hallucinations in patients taking zolpidem combined with SSRIs/SNRIs [5], but there is no published data on the toxic effects of supratherapeutic duloxetine combined with dexzopiclone, nor any clinical guidelines for the rescue of this drug combination toxicity. Notably, hallucinations with zolpidem are uncommon: in controlled trials of AMBIEN 10 mg at bedtime, <1% of adults with insomnia reported hallucinations, and in a pediatric study (6–17 years, ADHD-associated insomnia) hallucinations occurred in 7% of zolpidem-treated patients versus 0% with placebo; postmarketing reports exist, but their frequency cannot be reliably estimated [30]. Only isolated case reports describe the SSRI/SNRI-combination phenotype rather than cohort-level evidence [5]**.**Prior case-series reported supratherapeutic duloxetine ingestions involving cohorts of 63–106 patients [6–8]. Common manifestations included gastrointestinal upset, somnolence, tremor, tachycardia and hypertension; coma and hypotension occurred mainly in mixed-drug poisoning. Core management consisted of cardiorespiratory-QT monitoring and supportive care; activated charcoal may be considered within 6 h of ingestion, and no specific antidote is available [6–8].This case reports the successful emergency management of a young MDD patient with severe toxicity from co-ingestion of these two drugs, and summarizes the clinical characteristics, differential diagnosis points and standardized treatment strategies, aiming to provide a clinical reference for similar rare cases.

## Case presentation

A 23-year-old female (born March 1998) developed depressed mood and insomnia in August 2018 during her university studies in the United States and was diagnosed with depression and prescribed paroxetine hydrochloride (20 mg/day) and olanzapine (5 mg/day). She discontinued medication on her own one month later due to weight gain, with partial symptom relief. From November 2018 to April 2019, her symptoms worsened with intense suicidal ideation and wrist cutting; she was hospitalized in the US and diagnosed with MDD, post-traumatic stress disorder (PTSD) and borderline personality disorder. She received fluoxetine, olanzapine, prazosin and electroconvulsive therapy (ECT). This long-term dual low-dose antipsychotic maintenance regimen was prescribed by clinicians during her overseas treatment, and full original overseas medical records could not be retrieved. But she remained with suicidal ideation and returned to China in May 2019. No relevant medical or psychiatric history was reported in the family.

After returning to China, the patient refused hospitalization at a local psychiatric hospital, with a suicide risk assessment score of 22 points (high suicide risk) according to the suicide-prediction scale from reference [9]. Clinical manifestations included depressed mood, apathy, generalized soreness, reduced verbal output, low self-evaluation, hopelessness, partial insight, poor sleep, generalized myalgia, arthralgia, headache and glass-induced hand lacerations consistent with non-suicidal self-injury (NSSI, without intent to die). She was prescribed duloxetine hydrochloride enteric-coated tablets (60 mg/day), aripiprazole orally disintegrating tablets (5 mg/day) and quetiapine fumarate tablets (50 mg/day), with pain relief and improved sleep, but persistent suicidal ideation; her suicide risk score decreased to 3 points [9] (low suicide risk) one month later.

In November 2019, she returned to school and her medication was adjusted to escitalopram (10 mg daily), quetiapine (50 mg daily) and aripiprazole orally disintegrating tablets (5 mg daily), but depressive symptoms and suicidal ideation persisted. Due to recurrent generalized bone pain in December 2019, her medication was switched back to duloxetine hydrochloride enteric-coated tablets (60 mg/day), quetiapine (50 mg daily) and aripiprazole orally disintegrating tablets (5 mg daily) combined with weekly psychotherapy. Dexzopiclone was later added for refractory insomnia, with all medications kept by her mother to prevent overdose.

At 15:00 on February 18, 2021, the patient secretly ingested 4500 mg duloxetine hydrochloride enteric-coated tablets (30 mg/tablet) and 63 mg dexzopiclone tablets (3 mg/tablet). Since the patient had suicidal tendencies, her medications were normally kept by her mother. On the afternoon of the index day, the mother went out for an emergency errand, during which the patient took the medication without permission. The mother returned home 3.5 h later and discovered the incident. The patient was then rushed to our hospital. On admission at 19:00, she presented with delirium, unresponsiveness to verbal stimuli, coma, bilateral isocoric pinpoint pupils (diameter approximately 1 mm), sluggish light reflex, body temperature of 36.2 °C, reduced limb muscle tone and negative Babinski and Chaddock signs, and was immediately admitted to the intensive care unit for rescue.

### Emergency management and acute clinical course

After admission to the intensive care unit, the patient presented with restlessness and sinus tachycardia. Her blood pressure and blood glucose remained within normal ranges. No formal paper-based 12-lead ECG was recorded during acute resuscitation; only continuous bedside cardiac monitoring was performed. Serial 12-lead ECGs were obtained after clinical stabilization, showing basically normal findings as post-intoxication baseline cardiac status. She received oxygen inhalation (via nasal cannula, 5 L/min, preventive supportive measure; bedside pulse-oximetry showed SpO₂ > 96%, no clinical respiratory depression or hypoxia) and gastric lavage (using mineral water to reduce gastrointestinal drug absorption), which are the key emergency interventions for this comatose intoxication patient. At 20:15, the patient regained consciousness with bilateral isocoric pupils (diameter 2.5 mm) and normal light reflex. She was immediately given intravenous infusions including glucose and sodium chloride with potassium, high-dose vitamin B6 and C, and omeprazole for gastric mucosal protection. At 20:45, she had drowsiness and limb weakness with mild irritability, and was given 0.2 mg flumazenil intravenously to antagonize dexzopiclone toxicity. At 21:00, she developed two episodes of diarrhea and sinus tachycardia (100–120 beats/min), and was administered metoprolol sustained-release tablets (23.75 mg). The Hunter Serotonin Toxicity Criteria were applied before flumazenil administration; the patient did not exhibit pathognomonic findings suggestive of serotonin syndrome, so the risk of flumazenil-precipitated worsening of serotonergic toxicity was judged to be minimal [10]. A nucleic acid test was negative, and blood tests were basically normal (Table 1). Initial hemoglobin was 153 g/L (slightly above reference range), which decreased gradually following fluid resuscitation and toxicant elimination. Unfortunately, drug concentration detection was not performed due to lack of relevant equipment in our hospital and unavailability of external testing.

**Table 1:** Serial blood test results of the patient

Variables	Adult reference range	2021.02.18	2021.02.19	2021.02.20
Hematocrit (%)	35–45	46.4	43.0	41.4
Hemoglobin (g/L)	115–150	153	147	138
White-cell count (/μL)	3.5–9.5	7.54	13.30	10.27
Neutrophils (/μL)	1.8–6.3	4.87	11.51	6.76
Lymphocytes (/μL)	1.1–3.2	2.04	1.26	2.54
Monocytes (/μL)	0.1–0.6	0.57	0.51	0.85
Eosinophils (/μL)	0.02–0.52	0.04	0.01	0.09
Platelet count (/μL)	125–350	358	375	337
Prothrombin time (sec)	11–15	12.6	12.8	12.9
INR(International Normalized Ratio)	0.83–1.13	0.95	–	0.98
D-dimer (ng/ml)	<0.5	0.3	–	0.28
Sodium (mmol/L)	137–147	141.7	139.8	141.2
Potassium (mmol/L)	3.5–5.3	4.02	3.64	3.88
Chloride (mmol/L)	99–110	105.5	106	105.3
Carbon dioxide (mmol/L)	20.1–29.0	25.4	24.1	27
Urea nitrogen (mg/dl)	3–7.8	3.4	2.8	3.2
Creatinine (mg/dl)	40–120	48	45.2	52
Glucose (mg/dl)	3.8–6.1	5.07	6.64	4.80
Ferritin (μg/L)	13–150	–	–	–
Lactate dehydrogenase (U/L)	109–245	209	187	164
C-reactive protein (mg/L)	0–10	8.91	6.50	8.24
Creatine kinase (U/L)	22–270	54	44	43
Total protein (g/L)	65–85	76.6	72.1	66.4
Albumin (g/L)	40–55	47.2	44.2	42
Uric acid (μmol/L)	123–430	436.48	402.35	358.29
ALT (U/L)	7–40	29	29	23
AST (U/L)	10–35	22	27	20
Triiodothyronine (T3) (nmol/L)	0.99–2.73	–	–	1.31
Thyroxine (T4) (nmol/L)	78.38–157.4	–	–	136.82
Hypersensitive thyrotropin (mIU/L)	0.34–5.6	–	–	3.928
Free T3 (pmol/L)	3.28–7.42	–	–	4.82
Free T4 (pmol/L)	7.5–21.1	–	–	11.37
Thyroglobulin antibodies (IU/ml)	0–4	–	–	<0.10
Thyroid peroxidase antibody (IU/ml)	0–9	–	–	0.4
Troponin I (ng/mL)	0–0.023	–	–	0

At 02:00 on February 19, the patient developed sweating, visual hallucinations (numerous obvious color patterns in the visual field), hand tremors, headache, irritability, severe psychomotor agitation, 3–4 episodes of loose stools and urinary retention, with a heart rate of 90–100 beats/min and normal body temperature. She showed impaired sensorium with disorientation to time, place and person, fluctuating awareness and inconsistent attention. She received urinary catheterization and enhanced nursing care without specific drug treatment.

At 08:00 on February 19, her condition became stable, and she was transferred to the general ward for further treatment. At 11:00, recheck of blood routine, biochemical tests, abdominal color Doppler ultrasound, chest CT and brain MRI were normal, and ECG showed sinus tachycardia with ST-T segment changes. She received continuous rehydration, nutritional support, pantoprazole, enteral nutrition emulsion and topical eye drops for left corneal hemorrhage; the elevated white blood cell count was considered a physiological stress response and no specific treatment was given. During the daytime, her mental symptoms gradually improved, but she remained relatively non-verbal and slightly irritable. At 19:30, she left the hospital without authorization while wearing an indwelling urinary catheter bag when the nursing staff was inattentive. After being found 2 h later, she was retrieved and sent back to the ward, and she was given clonazepam (2 mg) and olanzapine orally disintegrating tablets (5 mg) for sedation, mood stabilization and anti-anxiety, and calmed down and fell asleep 1 h later with no clear recall of the incident the next day.

On the second hospital day, the patient had drowsiness and limb weakness, but could take enteral nutrition emulsion voluntarily; she recovered spontaneous urination after catheter removal, with significantly reduced sweating, mild nausea and one episode of loose stools. Her heart rate and blood pressure were normal, visual hallucinations improved, but hand tremors persisted, and blood test parameters showed significant improvement. She complained of generalized soreness at 16:39 and was given loxoprofen sodium (60 mg) and rabeprazole (10 mg) orally, with pain relief 3 h later.

On the third hospital day, visual hallucinations and nausea resolved, with normalized urination and defecation; mild finger tremors persisted and generalized body pain recurred. She cried due to severe pain and begged for duloxetine, and was given vitamin B6 (10 mg) orally as suggestive therapy (informed as a "painkiller"), with pain relief 30 min later. This therapy was repeated on February 23 for recurrent pain with consistent efficacy.

From the fourth hospital day, the patient's appetite gradually returned to normal; she could eat rice independently and began to communicate with family members, caregivers and medical staff, and even started to smile. From February 28, she took rice as the staple food supplemented with cake, juice and milk, with a basically normalized diet; intermittent body soreness persisted and was relieved by vitamin B6 suggestive therapy. Quetiapine (50 mg) was given at bedtime every night, and a psychologist conducted daily communication with her, but she still had depressed mood, pessimism and suicidal ideation. Per US FDA labeling, immediate-release quetiapine is only approved for schizophrenia and bipolar disorder. Clinical trial data support off-label low-dose (25–100 mg nightly) quetiapine to treat refractory insomnia in depressed patients, while it yields minimal improvement in core depressive symptoms [11]. This agent was given to the patient solely for persistent sleep disturbance.

On the 10th hospital day, considering presumed near-complete clearance of duloxetine and dexzopiclone inferred from clinical recovery and published pharmacokinetic data of duloxetine and dexzopiclone [6, 12, 13]. Under the guidance of a psychiatrist, the patient's medication was switched to lurasidone (20 mg/day) plus low-dose quetiapine (50 mg/day). Considering her prior history of bipolar-related mood disturbance, this regimen targeted residual depressive mood, emotional lability and prominent refractory insomnia after acute poisoning; close outpatient follow-up and medication supervision were arranged given the elevated safety risks of polypharmacy in this suicide-high-risk patient [14, 15]. Three days later, her generalized somatic pain resolved completely with normal sleep and no adverse reactions to the new regimen.

### Follow-up and outcome

The patient was discharged on March 2, 2021 (12 days after admission) with discharge advice including: (1) regular follow-up at the psychiatric outpatient department for dynamic medication adjustment and mental state evaluation; (2) routine blood routine and biochemical tests every 4 weeks to monitor organ function; and (3) continuous psychological intervention and family supervision to prevent self-injurious behavior and drug overdose.

During the subsequent outpatient treatment, the patient gradually tapered off lurasidone and quetiapine as advised by her physician. Routine blood tests, liver function and renal function monitored during outpatient follow-up were all normal.

At the latest follow-up in September 2025 (4.5 years post-poisoning), the patient reported that she had discontinued lurasidone and quetiapine, with normal verbal communication and social interaction ability. She was able to study, work and live independently, with no recurrent generalized somatic pain; occasional transient low mood was relieved by family communication, which she found helpful. No suicidal ideation or self-injurious behavior was observed, and she adhered to regular psychological treatment and psychiatric follow-up.

## Discussion

Severe toxicity following supratherapeutic co-ingestion of duloxetine and dexzopiclone remains rarely reported. Compared with reports of single-agent overdose, isolated duloxetine overdose predominantly produces sympathomimetic manifestations and rarely deep coma, whereas isolated dexzopiclone overdose mainly induces dose-dependent central sedation with minimal autonomic overactivity or hallucinations [4, 6]. Our case demonstrates a mixed toxic phenotype resulting from synergistic effects: dexzopiclone-related coma combined with SNRI-mediated sympathomimetic toxicity. Clinicians should be alert to potentially protracted clinical toxicity for such combined-ingestion scenarios [16].

The patient presented with a unique clinical profile of central nervous system depression, autonomic dysfunction and transient neuropsychiatric symptoms, posing significant diagnostic and therapeutic challenges. The key clinical insights from this case include accurate differential diagnosis of drug-induced adverse syndromes, cautious off-label use of low-dose flumazenil, and the efficacy of suggestive therapy and multidisciplinary collaboration in the recovery phase.

Poison control surveillance data show that co-ingestion of psychotropic agents frequently occurs in dexzopiclone self-poisoning events, and mixed polydrug exposure is also well documented among duloxetine overdose patients [6, 17]. Nevertheless, no individual published case report specifically describes combined duloxetine dexzopiclone intentional overdose, highlighting the clinical value of the present case.

Published toxicological data describe distinct intoxication patterns for duloxetine and the z-hypnotic dexzopiclone. Duloxetine overdose mainly induces sympathomimetic and serotonin-related features including tachycardia, tremor, sweating, hallucinations and gastrointestinal disturbance; supportive care is the mainstay, and no specific antidote is available [6]. Dexzopiclone overdose predominantly causes central nervous system depression, such as coma and reduced consciousness; flumazenil may be considered together with general supportive measures in appropriate clinical scenarios [4, 17]. In this case, coma and depressed consciousness matched typical dexzopiclone toxicity [4], while tachycardia, tremor, visual hallucinations and diarrhea corresponded to known duloxetine poisoning manifestations [6]. Thus the clinical picture reflected mixed-agent intoxication, and our interventions including gastric decontamination, supportive infusion and cautious low-dose flumazenil use followed established rescue principles for both intoxications [2].

### Differential diagnosis of drug-induced adverse syndromes

#### Neuroleptic malignant syndrome (NMS)

Neuroleptic malignant syndrome (NMS) is prioritized as the first-ranked differential diagnosis because it represents a life-threatening drug-induced emergency with substantial clinical overlap with psychotropic mixed-drug poisoning, including altered mental status, autonomic instability, tremor and muscular abnormalities. Delayed recognition may result in fatal complications, such as rhabdomyolysis, acute kidney injury and multi-organ dysfunction. Although NMS is classically induced by dopamine-blocking agents rather than SNRIs or Z-hypnotics, its phenotypic similarities to serotonin-related toxicity require primary exclusion in patients with altered mental status after psychotropic-drug exposure [18]. NMS is characterized by acute severe motor rigidity, hyperthermia, markedly elevated creatine phosphokinase (CPK) and leukocytosis [18], typically occurring within the first 10 days of neuroleptic treatment. In this patient, CPK levels remained normal throughout treatment, with no hyperthermia or limb rigidity (only reduced muscle tone); transient leukocytosis was a stress response to acute poisoning and resolved rapidly with supportive therapy, ruling out NMS.

#### Serotonin syndrome

Serotonin syndrome is caused by excessive central serotonin activity, with core features of clonus, hyperreflexia, mental status changes and autonomic dysfunction [18]. We further assessed our patient using the well-validated Hunter Serotonin Toxicity Criteria, which place emphasis on neuromuscular signs including clonus, hyperreflexia and tremor for diagnosing serotonin toxicity, rather than merely relying on non-specific mental-status changes. In this case, the patient had tremor, irritability, diarrhoea and visual hallucinations, but lacked key findings, such as clonus and robust hyperreflexia, which meant the patient did not fully satisfy diagnostic criteria for definite serotonin toxicity [10]. Although the patient had tremors, irritability, tachycardia and diarrhea, she lacked the pathognomonic signs of clonus and hyperreflexia. All symptoms resolved with drug clearance and supportive therapy without specific serotonin antagonist treatment, confirming the manifestations resulted from combined drug toxicity rather than serotonin syndrome.

#### Withdrawal syndrome

Long-term use of dexzopiclone may cause physical dependence, with withdrawal symptoms including agitation, sweating, tremors and hallucinations upon abrupt discontinuation [19]. However, the patient only used dexzopiclone intermittently before poisoning; her symptoms developed in the acute toxic phase (not post-discontinuation) and were exacerbated by residual duloxetine toxicity. Symptoms were rapidly relieved by clonazepam and olanzapine, with no recurrent withdrawal manifestations during follow-up, indicating only a minor withdrawal component in her clinical presentation.

#### Hyperactive delirium

The patient exhibited fluctuating awareness, disorientation, visual hallucinations, irritability, psychomotor agitation, and autonomic hyperactivity during acute intoxication, consistent with features of hyperactive delirium secondary to combined drug toxicity [20]. These neuropsychiatric manifestations fluctuated hourly and resolved completely after drug clearance, without core manic episode features (no expansive mood, grandiosity or pressured speech). This presentation was, therefore, attributed to toxic hyperactive delirium rather than primary manic disorder.

### Cautious off-label use of flumazenil in combined poisoning

Flumazenil is a specific benzodiazepine receptor antagonist that reverses the toxic effects of all Z-drugs including dexzopiclone, but its package insert contraindicates use in severe antidepressant poisoning [21]. Pathophysiologically, serotonin syndrome arises from excessive synaptic serotonin accumulation within the central nervous system. Benzodiazepines represent first-line symptomatic agents to control agitation and muscular hyperactivity in serotonin-toxicity states. By antagonizing GABAA-mediated central inhibitory tone, flumazenil can disinhibit neural excitability and theoretically precipitate or exacerbate serotonin-mediated neuroexcitation, even in partial serotonergic toxicity, which underpins this important contraindication [22].In this case, flumazenil (0.2 mg) was used exclusively to antagonize dexzopiclone toxicity, and was administered only after thorough gastric lavage with 10,000 mL of normal saline to remove unabsorbed duloxetine.

Importantly, flumazenil was administered only after ruling out concurrent serotonin syndrome; two-thirds of cardinal serotonin-toxicity symptoms were absent in this patient [10].Low-dose flumazenil partially relieved the patient's drowsiness and limb weakness caused by dexzopiclone, but did not improve tremors and irritability induced by residual duloxetine, confirming the synergistic toxic effects of the two drugs. This case validates that low-dose flumazenil can be used safely and effectively for dexzopiclone toxicity in duloxetine co-ingestion under close hemodynamic and neurological monitoring. Clinicians must exercise extreme caution with this off-label use: low doses only, after adequate gastrointestinal decontamination, with continuous monitoring for seizures and worsening antidepressant toxicity.

### Efficacy of suggestive therapy and psychological intervention

Somatic pain is a persistent and distressing symptom in MDD patients, and duloxetine is a first-line agent for depression-related somatic pain [23]. It should be emphasized that somatic pain comorbid with major depressive disorder represents a frequent clinical phenomenon rather than a discrete DSM-5-TR diagnosis or a standalone FDA-approved indication. Duloxetine carries formal FDA-approved indications for neuropathic pain and musculoskeletal pain, which confers therapeutic benefit for patients with MDD experiencing concurrent pain symptoms [2]. Discontinuation of duloxetine due to poisoning led to severe recurrent pain in this patient, who strongly associated pain relief with duloxetine use. Vitamin B6 administered as suggestive therapy achieved rapid and consistent pain relief, avoiding re-exposure to the offending drug and reducing the risk of recurrent toxicity. Prior to this suggestive-oriented intervention, thorough communication was conducted with the patient’s guardians and oral informed consent was obtained. Rigorous risk–benefit appraisal and full medical-record documentation were completed, and the measure was implemented in accordance with local institutional requirements and our hospital’s clinical standards. Given the patient’s persistent suicidal risk, reinitiating duloxetine would substantially raise the hazard of recurrent overdose; hence this short-term suggestive strategy was only used for acute pain management. Full truthful disclosure of this intervention was provided to the patient after her mental state stabilized, and she expressed no disagreement after explanation. It should be noted that clinical standards for such suggestive-based interventions differ across medical institutions. This non-pharmacological intervention has significant clinical value for drug poisoning patients with refractory somatic pain.

Psychological intervention and family involvement were critical to the patient’s long-term recovery. Daily psychotherapy targeted suicidal ideation and self-injurious behavior, while strict family supervision of medication storage prevented further overdose attempts. This case confirms that the integration of psychological support with pharmacotherapy is an essential component of treatment for MDD patients with drug overdose, consistent with previous studies [24] showing that psychological intervention improves treatment compliance and prognosis in such patients.

### Clinical lessons and medication safety recommendations

This rare case provides several critical clinical lessons for the management of MDD patients with somatic pain and insomnia, and the emergency treatment of drug co-ingestion toxicity:

1. **Strengthen medication safety for high-risk patients:** Young MDD patients (≤ 24 years) with suicidal ideation and self-injurious behavior are at high risk of drug overdose; all psychotropic medications should be stored and administered by a designated caregiver to prevent secret ingestion. Before the overdose event, this patient received combined psychotropic agents including duloxetine, aripiprazole, quetiapine and dexzopiclone. Such polypharmacy elevates the risk of both pharmacokinetic and pharmacodynamic drug-drug interactions, which may further increase the probability of adverse drug events. For high-risk MDD patients with suicidal ideation, clinicians should regularly review the necessity of each psychotropic medication and minimize unnecessary polypharmacy whenever clinically feasible.
2. **Cautious combination of SNRIs and Z-drugs:** It should be emphasized that risk profiles of SNRIs differ substantially between therapeutic-dose clinical use and overdose scenarios. While SNRIs constitute essential therapeutic agents for major depressive disorder and inappropriate discontinuation may produce considerable clinical harm. In contrast, the risk profile of isolated SNRI overdose is substantially milder than that of tricyclic antidepressants (TCAs) or monoamine oxidase inhibitors (MAOIs): isolated duloxetine overdose predominantly produces sympathomimetic features and, in published case series, no major complications or death [6, 13]. However, supratherapeutic duloxetine ingestion, especially in combination with Z-hypnotics or other CNS depressants, can still produce severe toxicity, coma and, rarely, fatality [13]**.** Individualized risk–benefit assessment should be performed for each psychotropic medication, instead of generalized lumped evaluation of multiple agents. Clinicians should weigh the benefits and risks of combining these drugs in young MDD patients, with close monitoring for suicidal ideation and prompt medication adjustment for non-responders.
3. **Standardized emergency management for combined poisoning:** For duloxetine-dexzopiclone co-ingestion, prompt thorough gastric lavage, adjuvant intravenous vitamin B6/C infusion, and continuous rehydration and organ protection are the core of rescue treatment. The patient manifested restlessness, clouded consciousness and poor oral intake during the acute poisoning episode. Intravenous isotonic saline was given for volume expansion to improve renal perfusion and glomerular filtration rate and facilitate urinary excretion of toxic substances. Vitamin B6 and vitamin C were used as adjuvant supportive interventions instead of specific antidotes. Vitamin B6 maintains the activity of hepatic detoxification-related co-enzymes, while vitamin C exerts antioxidant effects to alleviate oxidative stress induced by drug overdose. Neither vitamin B6 nor vitamin C directly catalyze or speed up the metabolic breakdown of duloxetine or dexzopiclone [25, 26].
4. **Accurate differential diagnosis is essential:** Drug-induced symptoms must be distinguished from NMS and serotonin syndrome based on core clinical features to avoid misdiagnosis and inappropriate treatment.
5. **Multidisciplinary collaboration for optimal prognosis:** Emergency medicine, neurology and psychiatry teams should collaborate closely to integrate acute poisoning rescue with long-term MDD management; psychological intervention and family involvement should be included in the entire treatment process.

### Study limitations

This study has a key limitation: the lack of blood and urine drug concentration detection, which prevented quantitative analysis of the toxicokinetic characteristics of duloxetine and dexzopiclone and their synergistic effects. Future studies should include drug concentration monitoring to further elucidate the toxicity mechanism of this rare drug combination. Additionally, long-term follow-up of more cases is needed to evaluate the long-term mental health and recurrence risk in patients with this severe poisoning.

## Conclusion

Severe toxicity following supratherapeutic co-ingestion of duloxetine and dexzopiclone remains rarely reported among young female patients with MDD. This report details the unique clinical manifestations, emergency rescue strategies and key differential-diagnosis points of this rare drug-combination toxicity. Standardized emergency management (thorough gastric lavage, high-dose vitamin infusion, cautious low-dose flumazenil use) and multidisciplinary intervention (symptomatic treatment, suggestive therapy, psychological support and family involvement) achieved an excellent clinical outcome, with the patient recovering completely and no recurrent suicidal ideation or self-injury at 4.5-year follow-up. These foundational supportive-care components are supported by published toxicology reviews and aggregate poisoning-cohort data, rather than derived exclusively from this single-case observation [27].This case fills a critical clinical gap in the management of duloxetine-dexzopiclone co-ingestion toxicity, and provides practical recommendations for clinicians: Importantly, low-dose flumazenil should not be regarded as part of standardized routine therapy for this mixed-intoxication scenario. Based on meta-analyses and official FDA prescribing information, flumazenil carries elevated risks of seizure and arrhythmia in patients with potential serotonergic toxicity [21, 22]. It should only be applied as a conditional, individualized rescue measure following rigorous pretreatment risk stratification. Comprehensive supportive care plus symptomatic benzodiazepine sedation represents the safer first-line alternative for this patient population, strengthen medication safety management for high-risk MDD patients; exercise caution in combining SNRIs and Z-drugs in young patients; adopt standardized decontamination and supportive therapy for combined poisoning with cautious off-label use of low-dose flumazenil; accurately distinguish drug-induced symptoms from NMS and serotonin syndrome; and integrate psychological intervention and family involvement into the entire treatment process. This case serves as an important clinical reference for emergency clinicians, neurologists, and psychiatric clinicians managing similar rare drug co-ingestion cases, and emphasizes the importance of medication safety and multidisciplinary care in the treatment of severe MDD with suicidal ideation and self-injurious behavior. According to the patient’s self-reported overseas medical records, she had a prior diagnosis of borderline personality disorder, which was not formally confirmed by structured diagnostic assessment during the current admission. Borderline personality disorder represents an independent risk factor for suicidal ideation and self-harm; impulsivity and emotional dysregulation associated with this condition further raise the risk of intentional self-poisoning [28, 29].

## Supplementary Information

Additional file 1.

## Data Availability

No datasets were generated or analysed during the current study.
