# Supplementary material for: Severe toxicity following supratherapeutic co‑ingestion of duloxetine and dexzopiclone in a young patient with major depressive disorder: a case report
Source: J Med Case Rep. 2026 Nov 6;20:709. doi: 10.1186/s13256-026-06736-y (PMC13746538; doi:10.1186/s13256-026-06736-y)
Supplement: Supplementary file 1 [file 13256_2026_6736_MOESM1_ESM.pdf]

**Topic**	**Item No.**	**Checklist item**	**Reported on Page / Section in revised manuscript**
Title	1	Title contains words "case report" and highlights primary focus of the case	Page 1, Lines 1-3<br>Title section
Keywords	2	2-5 keywords identifying key features of this case	Page 2, Lines 30-31<br>Keywords section
Abstract	3a	Background: Describe what is unique about this case and its contribution to medical literature	Page 2, Lines27-29<br>Abstract second paragraph
	3b	Patient's chief complaints and important clinical findings	Page 1, Lines 15-26<br>Abstract first paragraph
	3c	Primary diagnoses, interventions and major clinical outcomes	Page1-2, Abstract first paragraph
	3d	Conclusion: Core take-home messages from this case (abstract conclusion)	Page 2, Lines27-29<br>Abstract second paragraph<br>Page 19, Lines 379-386<br>Page 20, Lines 389-405
Introduction	4	Brief background, relevant literature, rationale for reporting this case	Page 3, Lines 49-52、58-66,<br>Section 1 Introduction
Patient information	5a	De-identified demographics: age, gender, ethnicity, occupation	Page 4, Lines 68-69<br>Section 2 Case Presentation opening sentence
	5b	Patient's chief presenting symptoms	Page 3-4, Section 2 Case Presentation: admission clinical manifestations after overdose

Topic	Item No.	Checklist item	Reported on Page / Section in revised manuscript
			Ingestion
	5c	Medical / family / psychosocial history, comorbidities	Page 3-5, Section 2 Case Presentation: past psychiatric, family and psychosocial history
	5d	Previous relevant interventions and their outcomes	Page 3-5, Section 2 Case Presentation: prior pharmacotherapy and overseas treatment history
Clinical Findings	6	Key physical examination, vital signs and clinical findings	Page 5, Lines 104-107, Section 2 Case Presentation: admission vital signs and physical examination findings
Timeline	7	Important dates and chronological sequence of clinical events during care episode	Page 5, Lines 98-103<br>Page 6-7, Lines 111-154<br>Page 8, Lines 155-176<br>Page 9, Lines 177-198<br>full Section 2 Case Presentation (2, 2.1, 2.2): poisoning onset, hospital course, discharge
Diagnostic Assessment	8a	Diagnostic methods: physical exam, laboratory tests, imaging examinations	Page 5, Lines 104-107<br>Page 6, Lines 111-113、127-131<br>Page 7, Lines 138-141<br>Page 10, Line 199 (Table 1)
	8b	Diagnostic difficulties / limitations encountered	Page 6, Lines 130-131<br>Page 19, Lines 371-377<br>Subsection 3.5 Study Limitations (lack of drug concentration testing)

**Topic**	**Item No.**	**Checklist item**	**Reported on Page / Section in revised manuscript**
	8c	Differential diagnoses considered and reasoning	Page 12-14, Subsection 3.1 Differential Diagnosis of Drug-Induced Adverse Syndromes
	8d	Prognostic features (where applicable)	Page 9, Lines 190-198 Subsection 2.2 Follow-up and Outcome
Therapeutic Intervention	9a	Types of interventions: pharmacologic / supportive care etc.	Page 5-6, Lines 114-116、119-122<br>Page 7-8,Lines 136-137、148-150<br>Lines 156-157、159-162、165-173<br>Subsection 2.1 Emergency Management and Acute Clinical Course
	9b	Dose, route, timing, duration of each intervention	Page 5-8, Subsection 2.1 Emergency Management and Acute Clinical Course<br>Page 5-6, Lines 114-116、119-122<br>Page 7, Lines 137、148-150<br>Page 8, Lines 156-157、160-162、167-173
	9c	Any changes to interventions and rationale for adjustment	Page 8-9, Lines 174-177 Subsection 2.1: medication optimization at hospital day 10
Follow-up and Outcomes	10a	Outcomes assessed by clinician and patient	Page 9, Lines 185-198; Subsection 2.2 Follow-up and Outcome (clinical outcome at discharge and 4.5-year follow-up)<br>Line 199 (Table 1)

**Topic**	**Item No.**	**Checklist item**	**Reported on Page / Section in revised manuscript**
	10b	Key follow-up laboratory / examination results	Page 9-10, Line 190-192； 198 Line 199 (Table 1)<br>Subsection 2.2 Follow-up and Outcome: outpatient laboratory monitoring results
	10c	Treatment adherence and tolerability	Page 5, Lines 90-97<br>Page 9, Lines 190-192、198<br>Subsection 2.2 Follow-up and Outcome: outpatient medication tapering and psychiatric follow-up
	10d	Adverse events or unexpected clinical occurrences	Page 6, Lines 132-137<br>Page 7, Lines 145-147<br>Subsection 2.1 Emergency Management and Acute Clinical Course (delirium, hallucinations, agitation, self-leave hospital episode)
Discussion	11a	Strengths and limitations of this case management	Strengths：P2 Lines 27-29;<br>Page 19 Lines379-386;<br>Page 20 Lines402-405<br>Limitations: Page 19, Lines 371-377<br>Subsection 3.5 Study Limitations
	11b	Comparison with existing published literature	Page 11-12, Lines 201-230<br>Discussion opening paragraphs (literature comparison, before subsection 3.1 Differential Diagnosis)
	11c	Rationale for conclusions, cause-effect inference	Page 12-19, Lines 231-370<br>Discussion subsections 3.1-3.4 (differential diagnosis and causal toxicological inference)

Topic	Item No.	Checklist item	Reported on Page / Section in revised manuscript
	11d	Key clinical take-home lessons	Page 19-20, Lines 389-405 Section 4 Conclusion
Patient Perspective	12	Patient's own experience / viewpoint (if available)	Not applicable; dedicated patient narrative statement was not collected; all descriptions derived from medical records
Informed Consent	13	Written informed consent for publication obtained from patient	Page 21, Lines 418-422. Consent to publish , Declarations section, Consent to publish paragraph

Notes:

1. Page and section references refer to revised manuscript (2026-10-06 version (2nd-round revision)).
2. Item 12 (Patient perspective): Not applicable, no dedicated patient narrative was collected for publication. All clinical descriptions are extracted from hospital medical records.
3. Written informed consent for publication has been obtained from the patient and stored within hospital medical archives, and can be provided to the editorial office upon reasonable request.

This checklist is adapted from the official CARE-Statement 2013 reporting guideline (https://www.care-statement.org/checklist/)
